# Effectiveness of pharmacist-delivered vaccination recommendations for herpes zoster: a systematic review

**DOI:** 10.3389/fpubh.2026.1884479

**Published:** 2026-08-17

**Authors:** Ewa Kuczwalska, Anna Maria Dworakowska, Magdalena Skarżyńska, Magdalena Bujalska-Zadrożny

**Affiliations:** 1 Department of Pharmacotherapy and Pharmaceutical Care, Faculty of Pharmacy, Medical University of Warsaw, Warsaw, Poland; 2 Clinical Trials Department, Center of Hearing and Speech, Kajetany, Poland; 3 Institute of Sensory Organs, Kajetany, Poland

**Keywords:** community pharmacy, herpes zoster, immunization uptake, pharmacist, systematic review, vaccination

## Abstract

**Introduction:**

Herpes zoster (HZ) remains an important cause of morbidity among older adults despite the availability of effective vaccines. Recommendations from healthcare professionals are among the strongest determinants of adult vaccination uptake, and community pharmacists are increasingly involved in vaccination recommendations.

**Objectives:**

To systematically review and critically appraise the evidence on the effectiveness of pharmacist-delivered recommendation strategies in increasing confirmed HZ vaccination uptake and recombinant zoster vaccine (RZV) series completion.

**Patients and methods:**

PubMed, Embase, and the Cochrane Library were searched from database inception to October 14, 2025. Studies evaluating pharmacist-delivered recommendation interventions aimed at increasing HZ vaccination were included if they reported quantitative outcomes for confirmed vaccination uptake or RZV series completion. Data on study characteristics, interventions, and vaccination outcomes were extracted. Risk of bias was assessed using RoB 2 and ROBINS-I.

**Results:**

Nine studies met the eligibility criteria for the primary synthesis of confirmed vaccination outcomes. Eight evaluated ZVL (Zostavax), whereas only one specifically assessed RZV (Shingrix) series completion. Seven studies were conducted in the United States and used predominantly non-randomized designs. Settings ranged from single community pharmacies to networks of 159 pharmacies. Positive effects on confirmed HZ vaccination uptake or RZV series completion were reported in five studies. The strongest evidence came from a cluster randomized controlled trial involving 16 pharmacies, in which automated telephone recommendations increased vaccination rates from 0.72 to 2.60% (OR 3.69; 95% CI 2.64–5.15). Additional positive findings were observed for personalized outreach and pharmacist telephone follow-up aimed at improving RZV series completion. Three studies reported no improvement or decreased HZ vaccination rates. The overall certainty of evidence was limited by methodological weaknesses: six of eight non-randomized studies were rated as having serious risk of bias, and only one randomized trial was available.

**Conclusion:**

Pharmacist-delivered recommendation strategies may improve HZ vaccination uptake and RZV series completion, particularly when implemented through proactive patient outreach. However, the current evidence base remains limited by the predominance of non-randomized studies and substantial risk of bias. Well-designed randomized and quasi-experimental studies are needed to determine the independent effectiveness of pharmacist-delivered vaccination recommendations.

## 1 Introduction

Herpes zoster (HZ), commonly known as shingles, is caused by reactivation of the varicella-zoster virus (VZV) and predominantly affects adults aged 50 years and older. The estimated lifetime risk of developing HZ is approximately 30%, and complications—most notably postherpetic neuralgia (PHN)—occur in 10–13% of patients aged 60 years and older, resulting in significant morbidity and reduced quality of life (1, 2).

Two vaccines are currently available for the prevention of HZ: the live attenuated zoster vaccine (ZVL; Zostavax, Merck), which received United States Food and Drug Administration approval in 2006 and was initially recommended for adults aged 60 years and older, and the recombinant zoster vaccine (RZV; Shingrix, GSK), approved in 2017, which is now the preferred vaccine recommended by the Advisory Committee on Immunization Practices (ACIP) for adults aged 50 years and older (3, 4). RZV offers superior efficacy (84–97% against HZ, depending on age group) and is administered as a 2-dose intramuscular series (3).

Despite vaccine availability, HZ vaccination rates remain suboptimal in many countries. In the United States, coverage among adults aged 60 years and older was estimated at approximately 34.5% (4). Barriers to uptake include low patient awareness, insufficient provider recommendations, cost, and vaccine access issues (5).

Community pharmacists are increasingly recognized as key contributors to adult immunization programs. Their high accessibility—nearly 91.7% of Americans live within 5 miles of a community pharmacy—and extended operating hours position them well to identify vaccination-eligible patients, provide education, and administer vaccines (6). A healthcare professional’s recommendation is consistently identified as one of the strongest predictors of patient vaccination (7, 8). Pharmacist-delivered vaccination recommendations therefore represent a potentially scalable intervention for improving HZ vaccination uptake.

Several prior systematic reviews have examined the broader role of pharmacists in adult immunization; however, none has focused specifically on the effectiveness of recommendation strategies as an isolated intervention for HZ vaccination. This systematic review aims to fill this gap by synthesizing and critically appraising the available evidence on the effectiveness of pharmacist-delivered recommendation strategies in increasing HZ vaccination uptake and series completion.

## 2 Patients and methods

### 2.1 Search strategy

This systematic review was conducted in accordance with the PRISMA 2020 guidelines for reporting systematic reviews (9). A systematic search was conducted in PubMed, Embase, and the Cochrane Library from database inception to October 14, 2025. The search strategy combined terms related to pharmacists and pharmacies with terms related to herpes zoster and vaccination (pharmacist OR community pharmacy OR pharmaceutical care OR pharmaceutical service) AND (herpes zoster OR shingles OR postherpetic). Detailed database-specific strategies are provided in Supplementary Tables S1–S3. Reference lists of included studies and relevant reviews were also examined.

### 2.2 Eligibility criteria

Studies were eligible for inclusion if they met the following criteria:

#### 2.2.1 Population

Adults (aged ≥18 years) eligible for HZ vaccination.

#### 2.2.2 Intervention

Any pharmacist-led or pharmacist-delivered recommendation strategy aimed at increasing HZ vaccination uptake, including but not limited to verbal recommendations, automated telephone messages, personalized written communications, motivational interviewing, or technology-supported proactive recommendation systems.

#### 2.2.3 Vaccine type

Studies were eligible if they investigated HZ vaccination using any licensed vaccine, including the live attenuated zoster vaccine (ZVL; Zostavax) or the recombinant zoster vaccine (RZV; Shingrix). Given that the transition from ZVL to RZV occurred during the study period covered by this review (ZVL recommended 2006–2017; RZV preferred from October 2017), both vaccine types were considered eligible, and the vaccine type investigated in each included study is specified in the results.

#### 2.2.4 Outcomes

The primary outcome was confirmed actual HZ vaccination uptake or series completion (doses administered, as documented in pharmacy records, immunization information systems, or dispensing databases). Studies were required to report quantitative vaccination rates or counts. Studies reporting only vaccination intention, commitment, readiness to vaccinate, or vaccine assessment outcomes without confirmed vaccination uptake were excluded.

#### 2.2.5 Setting

Community pharmacy settings or non-hospital community settings where pharmacists delivered the intervention.

Excluded were studies evaluating only vaccine administration without a recommendation component, studies conducted in hospital inpatient settings, case reports, and reviews.

### 2.3 Study design classification

Study designs were categorized as follows: cluster randomized controlled trial (cRCT); uncontrolled pre–post study (a before–after design in which vaccination rates measured during an intervention period are compared with rates from a historical control period—typically the same calendar period in the prior year—with no concurrent external control group); comparative cross-sectional study; retrospective cohort study; and prospective descriptive/implementation study. The pre–post design is distinct from a prospective implementation study with a concurrent control arm in that it relies entirely on historical comparators and therefore cannot exclude the influence of secular trends, seasonal variation, or co-occurring changes in policy, vaccine availability, or healthcare delivery. Studies embedded within larger multicomponent demonstration projects were classified according to their primary design and noted as part of a multicomponent intervention.

### 2.4 Data extraction

All records were imported into Rayyan (Rayyan Systems Inc., Cambridge, MA, United States) for deduplication and screening. After removal of duplicates, titles and abstracts were independently screened by two reviewers using predefined criteria. Full-text articles were assessed for eligibility, and disagreements were resolved by discussion or consultation with a third reviewer. Reference lists of included studies were screened to identify additional relevant publications.

Data were independently extracted by two reviewers using a standardized extraction form. Extracted variables included: study design, country, setting (number and type of pharmacies or sites), target population, type of pharmacist-delivered recommendation, other intervention components, vaccine type assessed, primary and secondary outcomes, and study results.

### 2.5 Risk of bias assessment

Risk of bias was assessed independently by two reviewers. The Cochrane Risk of Bias 2 (RoB 2) tool (10) was applied to the single randomized controlled trial (11). The Risk of Bias in Non-randomized Studies of Interventions (ROBINS-I) tool (12) was applied to all 8 non-randomized studies (13–20). The ROBINS-I domains assessed were: (1) bias due to confounding; (2) bias in selection of participants into the study; (3) bias in classification of interventions; (4) bias due to deviations from intended interventions; (5) bias due to missing data; (6) bias in measurement of outcomes; and (7) bias in selection of reported results. Overall risk of bias for each non-randomized study was rated as low, moderate, serious, or critical. Results of the risk of bias assessment are reported in the Results section.

### 2.6 Synthesis

Due to substantial heterogeneity in study designs, settings, intervention types, and outcome measures, a meta-analysis was not appropriate. Results are presented in a narrative synthesis, structured according to outcome type (actual vaccination uptake; vaccination intention or commitment).

## 3 Results

### 3.1 Study selection

The systematic search identified 835 records. After deduplication, 577 unique records were screened by title and abstract by two independent reviewers (EK and MS) using PICOS-based criteria. Discrepancies were resolved by consensus or, when necessary, by a third reviewer (AD). Fifty-one full-text articles were assessed for eligibility. Nine studies met the inclusion criteria and were included in the qualitative synthesis (11, 13–20). Three studies identified during full-text review reported only vaccination intention, commitment, readiness, or vaccine assessment outcomes and did not meet the predefined outcome criterion of confirmed HZ vaccination uptake. These studies were excluded from the final synthesis (21–23).

**Figure 1:**
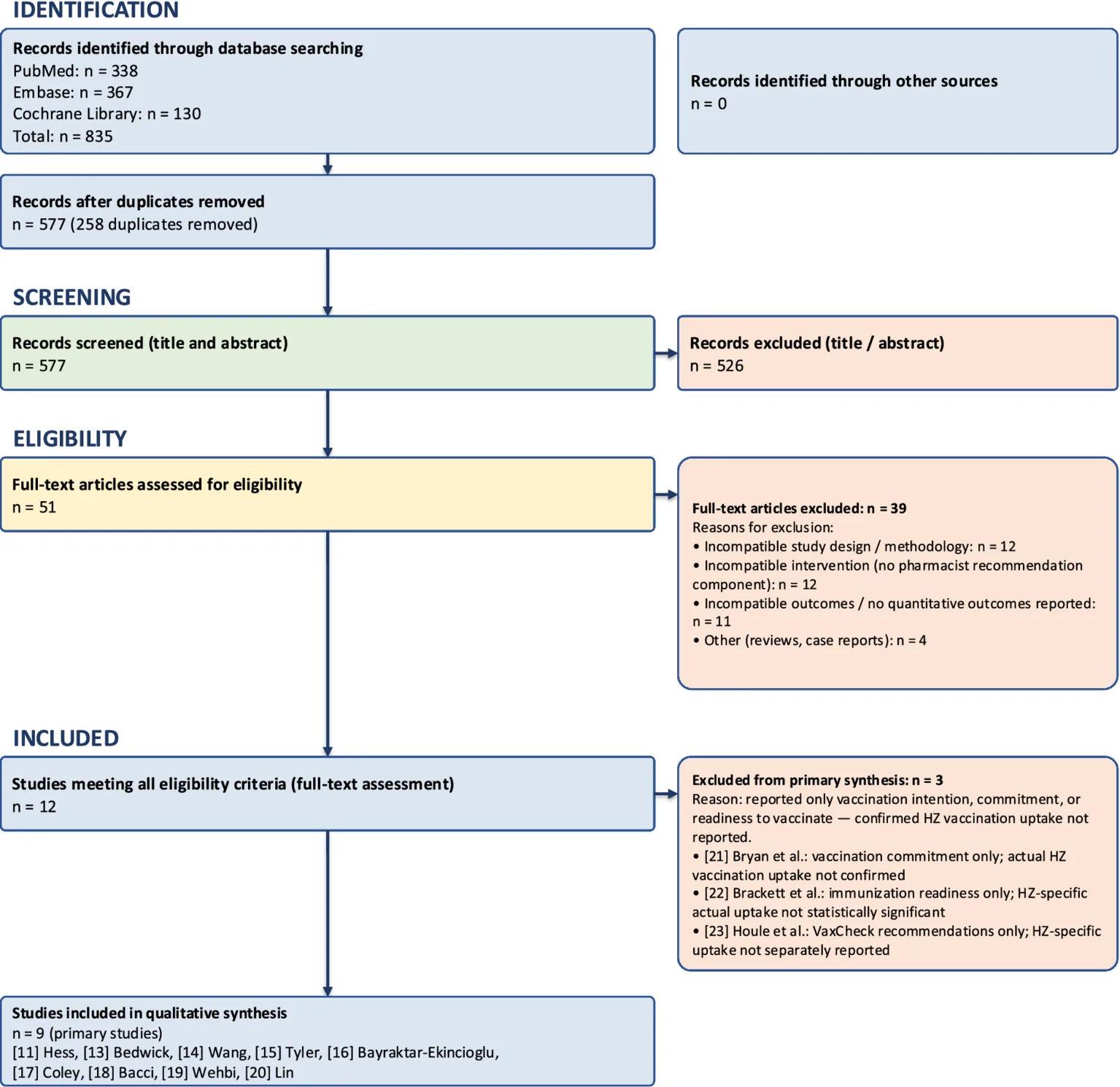
PRISMA 2020 flow diagram of the study selection process. Twelve studies met eligibility criteria at full-text assessment. Three studies (21–23) were assessed in full but excluded from the primary synthesis because they did not report confirmed HZ vaccination uptake as an outcome (reporting only vaccination intention, commitment, or readiness to vaccinate). Nine studies were included in the qualitative synthesis (11, 13–20).

The study selection process is summarized in the PRISMA flow diagram (Figure 1).

### 3.2 Characteristics of included studies

Nine studies met the inclusion criteria and were included in the qualitative synthesis (11, 13–20). Eight studies (89%) were conducted in the United States (11, 13–15, 17–20) and one in Türkiye (16).

Study settings varied considerably. Across the nine included studies, interventions were implemented in 363 unique pharmacies or community sites. This estimate accounts for the fact that the eight pharmacies reported by Lin et al. (20) were a subset of the 70 pharmacies included in the study by Bacci et al. (18) (including one study conducted in five social and solidarity centers). Two studies were conducted in single independent community pharmacies (13, 14)—of which (14) used 3 pharmacies]. The scale ranged from single independent pharmacies to large multi-site networks: 3 pharmacies (14), 8 pharmacies (20), 10 pharmacies (15), 16 pharmacies (11), 70 pharmacies (18), 99 pharmacies (17), and 159 pharmacies (19). Bayraktar-Ekincioglu et al. (16) conducted their study in five social and solidarity centers coordinated by the municipality not pharmacy settings in Ankara, Türkiye, which represents a distinct non-pharmacy community setting.

Study designs were heterogeneous. There was one cluster randomized controlled trial (11); six uncontrolled pre–post studies (13, 14, 17–20); one retrospective cohort study (15); and one prospective study conducted in a community (non-pharmacy) setting (16). The study by Lin et al. (20) was a subset of Project VACCINATE, the same demonstration project reported by Bacci et al. (18), evaluating two training strategies within 8 of the 70 participating pharmacies.

Target populations were adults aged ≥60 years in most studies focusing on HZ-eligible patients (11, 13, 14), adults ≥50 years in Türkiye (16), and adults of various ages eligible for any of multiple targeted adult vaccines in the larger implementation studies (17–20). The total number of participants varied widely: from approximately 600 patients contacted by phone in one independent pharmacy (13) to over 1,000,000 patients targeted in the 159-pharmacy implementation project (19).

Regarding vaccine type: studies conducted before 2017 exclusively investigated ZVL (Zostavax), as RZV was not yet available (11, 13–17). Tyler et al. (15) explicitly evaluated RZV (Shingrix) series completion (first dose June 2018–May 2019). Bacci et al. (18) and Wehbi et al. (19) conducted their projects between September 2016 and September 2017, spanning the period of both ZVL availability and RZV approval (October 2017), and did not specify the vaccine type for each individual HZ vaccination. Lin et al. (20) conducted their project from September 2016 onwards and did not specify vaccine type. The characteristics of all included studies are summarized in Table 1.

**Table 1:** Characteristics and outcomes of included studies.

Study (ref)	Setting (*N* pharmacies/sites; country)	Study design	Population	Intervention	Vaccine type	Primary comparator	Outcome measure	Key result (HZ-specific)	RoB
Bedwick et al., 2017 (13)	1 independent pharmacy, United States	Pre–post	Adults ≥60 yrs.; ~600 called	Automated telephone message (owner-recorded, 3×)	ZVL (Zostavax)	Same 3-month period, prior year	Number of HZ vaccinations	25 (intervention) vs. 16 (control); phone call = most cited reason for vaccination (16/18 callers influenced)	Serious (ROBINS-I)
Hess, 2013 (11)	16 grocery-chain pharmacies (8 int., 8 ctrl), United States	Cluster RCT	Adults ≥60 yrs.; *n* = 5,599 (int.), *n* = 6,383 (ctrl)	30 s automated telephone message (3 months)	ZVL (Zostavax)	No call (control pharmacies)	HZ vaccination rate	2.60% vs. 0.72% (OR 3.69; 95% CI 2.64–5.15; *p* < 0.001)	Low (RoB 2)
Wang et al., 2013 (14)	3 independent pharmacies, United States	Pre–post	Adults ≥60 yrs.; *n* = 59 (ctrl), *n* = 193 (int.)	Multicomponent: newspaper press release + prescription-attached flyer + personalized mailed letter	ZVL (Zostavax)	Prior 4-week period (Feb 2008)	HZ vaccination rate	0.37% (59/16,121) → 1.20% (193/16,062); *p* < 0.0001	Serious (ROBINS-I)
Tyler et al., 2021 (15)	10 Walgreens pharmacies, Mississippi, United States	Retrospective cohort	Patients receiving 1st RZV dose (June 2018–May 2019); *n* = 271	Pharmacist telephone call (SHINGRIX call list) as reminder for 2nd RZV dose	RZV (Shingrix)	No pharmacist phone call	RZV series completion (2nd dose received)	63.8% (44/69) vs. 46.0% (93/202); *p* < 0.05. Cost of first dose not significantly associated with return.	Moderate (ROBINS-I)
Bayraktar-Ekincioglu et al., 2022 (16)	5 social/solidarity centers (NOT pharmacies), Ankara, Türkiye	Prospective study (pre–post with 1-year follow-up)	*n* = 155; mean age 68.72 yrs.; 72.9% male; not vaccinated at baseline	Individual pharmacist educational session (~20–30 min) on HZ and PN vaccines + booklet	ZVL (live attenuated; NOT reimbursed in Türkiye at time of study)	1-year telephone follow-up (no control group)	Self-reported HZ vaccination at 1-year follow-up	Before: 12.7% knew of HZ vaccine. After session: 51.6% willing. At 1-year: only 0.8% actually vaccinated for HZ (5.7% for PN).	Serious (ROBINS-I)
Coley et al., 2020 (17)	99 Giant eagle pharmacies, western Pennsylvania, United States	Pre–post (demonstration project)	Adults insured in single managed care org.; HZ criterion: ≥60 yrs	Multicomponent: automated notifications (receipt/bag alerts) + telephonic outreach + motivational interviewing (face-to-face and telephone) + IIS registry interface	ZVL (Zostavax; 2016-2017)	Prior 12-month period (Sept 2015–Aug 2016)	Number of vaccines administered (4 types)	Overall: +33% vaccinations. Influenza: +45%, Pertussis: +31%, Pneumococcal: +7%. HZ: −5% decrease.	Serious (ROBINS-I)
Bacci et al., 2019 (18)	70 pharmacies (40 Bartell + 30 QFC), western Washington, United States	Pre–post observational (demonstration project)	Adults with potential age/medical indication for 4 vaccines	ImmsLink IIS interface + vaccine forecasting + proactive pharmacist recommendation + value-based payment (12-pharmacy subgroup)	ZVL (Zostavax; 2016-2017)	Prior 12-month period (Sept 2015–Aug 2016)	Number of vaccines administered (4 types)	Overall: +15% total vaccines. HZ: “appeared to be unaffected”—no significant HZ increase reported.	Serious (ROBINS-I)
Wehbi et al., 2019 (19)	159 Hy-Vee pharmacies (129 Iowa + 30 Nebraska), United States	Pre–post observational (demonstration project)	Adults ≥19 yrs.; ~1,021,170 targeted	ImmsLink bidirectional IIS platform + personalized on-screen recommendations + promotional materials (newspapers, flyers, personalized mail)	ZVL predominantly; RZV possibly (project Oct 2016–Sept 2017)	Prior 12-month period (2015-2016)	Number of HZ vaccinations (registry-linked counts)	HZ: +12% (5,146 → 5,769). Influenza: +37%. Pertussis: +74%. Pneumococcal: −16%.	Moderate (ROBINS-I)
Lin et al., 2018 (20)	8 QFC pharmacies, Seattle, Washington, United States (subset of Bacci, 2019 (18))	Pre–post (training comparison: whole-staff vs. train-the-trainer)	Adults ≥18 yrs.; pharmacists and technicians trained	ImmsLink + proactive patient engagement + two training strategies (whole-staff or train-the-trainer)	ZVL (Zostavax; 2016)	Same 3-month period, prior year	Total vaccines administered (4 types combined)	Total vaccines: +12.6% (whole-staff) and +15.2% (train-the-trainer). HZ-specific outcomes not isolated.	Serious (ROBINS-I)

All data verified against original published articles. RoB, risk of bias; ZVL, live attenuated zoster vaccine (zostavax); RZV, recombinant zoster vaccine (shingrix); IIS, immunization information system; MI, motivational interviewing; PN, pneumococcal vaccine; HZ, Herpes zoster; cRCT, cluster randomized controlled trial.

### 3.3 Risk of bias assessment

The single cluster RCT (11) was assessed using RoB 2 (10) and was rated as low risk of bias overall. Cluster randomization was appropriately implemented, allocation was concealed, all pharmacy cohorts received identical promotional resources and vaccine supplies, and pharmacists were trained uniformly. The main limitation was that patients and pharmacists were not blinded to the intervention, which is inherent to this type of study.

The non-randomized studies were assessed using ROBINS-I (12). Six of 8 non-randomized studies were rated as serious risk of bias (13–18, 20), and 2 were rated as moderate risk of bias (15, 19). No study was rated as low or critical risk of bias.

The most prevalent sources of bias were: (1) absence of a concurrent external control group, present in 7 of 8 non-randomized studies (13, 14, 16–20), with historical data from the prior year used as the comparator; (2) confounding due to co-occurring changes in vaccine availability (transition from ZVL to RZV), reimbursement policies, and seasonal vaccine promotion campaigns; (3) co-intervention bias in studies embedded within large multicomponent system-level demonstration projects (17–20), where it was not possible to isolate the contribution of the pharmacist recommendation from other simultaneously implemented changes (e.g., IT systems, patient reminders, registry interfaces, staff training, and motivational interviewing); and (4) outcome measurement bias, particularly in studies reporting only self-reported outcomes (16). The retrospective cohort design of Tyler et al. (15) and the large scale of Wehbi et al. (19) with 159 pharmacies and registry-linked data were considered to reduce some confounding risk relative to smaller pre–post studies, and both were rated as moderate risk of bias. A summary risk-of-bias assessment is presented in Supplementary Table S4.

### 3.4 Characteristics of pharmacist-delivered recommendation strategies

Pharmacist-delivered recommendation strategies varied substantially across the 9 included studies and were categorized into five types:

1. Automated telephone messages: pharmacist-recorded or chain-generated prerecorded calls sent to eligible patients (11, 13).
2. Personalized written or verbal recommendations: including personalized letters mailed to patients identified through pharmacy profiles, prescription-attached flyers, and local newspaper press releases (14).
3. Telephone follow-up for series completion: pharmacist-initiated calls to patients who had received their first RZV dose to remind and encourage return for the second dose (15).
4. Technology-supported proactive recommendation systems: integration of immunization information system (IIS) interfaces with pharmacy dispensing systems (ImmsLink) enabling pharmacists to generate personalized vaccine forecasts and proactively recommend indicated vaccines during routine dispensing workflow (17–20).
5. Individual educational sessions in non-pharmacy community settings: structured face-to-face educational sessions delivered by pharmacists in social and solidarity centers, addressing HZ disease burden, vaccine efficacy, and reimbursement status, supplemented by written informational materials (16).

Recommendations constituted the primary intervention in three of the nine studies (11, 13, 15), whereas the remaining six evaluated recommendations as part of multicomponent interventions (14, 16–20).

### 3.5 Quantitative effects on herpes zoster vaccination uptake

Five of the nine studies (55.6%) reported positive effects on HZ vaccination uptake or RZV series completion (11, 13–15, 19).

The most methodologically robust evidence came from the cluster RCT by Hess (11), which demonstrated that automated telephone messages delivered monthly for 3 months to patients of 16 community pharmacies (grocery-store chain, Georgia and Tennessee) resulted in HZ vaccination rates of 2.60% (146 vaccinations, *n* = 5,599 patient cohort) versus 0.72% (46 vaccinations, *n* = 6,383 patient cohort) in the control group, representing a statistically significant difference (OR 3.69; 95% CI 2.64–5.15; *p* < 0.001). The vaccine studied was ZVL (Zostavax), which was the only approved HZ vaccine at the time of the study (2007).

In the pre–post study by Bedwick et al. (13), automated telephone messages (recorded by the pharmacy owner) sent to approximately 600 patients aged ≥60 years over 3 months in a single independent community pharmacy were associated with an increase in HZ vaccinations from 16 during the control period (same 3-month period of the prior year) to 25 during the intervention period (ZVL; Zostavax). The phone call was cited as the most common reason for vaccination: 18 of 24 surveyed patients reported receiving the call, and 16 of those 18 stated it directly influenced their decision to vaccinate.

Wang et al. (14) conducted a multicomponent intervention (press release, prescription-attached flyers, and personalized mailed letters) in three independent community pharmacies in Tennessee, targeting patients aged ≥60 years. Using ZVL, the HZ vaccination rate increased from 0.37% (59/16,121) during the 4-week control period (February 2008) to 1.20% (193/16,062) during the 4-week intervention period (March 2008) (*p* < 0.0001). More patients attributed their vaccination decision to pharmacist-driven interventions than to physician recommendation.

Tyler et al. (15) conducted a retrospective cohort study across 10 Walgreens pharmacies in Mississippi, evaluating the impact of pharmacist telephone follow-up on RZV (Shingrix) series completion among patients who had received their first dose between June 2018 and May 2019. Of 271 analyzed patients, 44 of 69 (63.8%) who spoke with a pharmacist returned for their second RZV dose, compared with 93 of 202 (46.0%) who did not receive the pharmacist phone call (*p* < 0.05). The cost of the first dose to the patient was not significantly associated with second-dose return (OR 0.6703; 95% CI 0.4153–1.082).

Wehbi et al. (19) implemented a bidirectional registry-linked technology platform (ImmsLink) in 159 Hy-Vee pharmacies across Iowa (*n* = 129) and Nebraska (*n* = 30), enabling pharmacists to access patient immunization histories and generate personalized vaccine recommendations. Over the 12-month demonstration period (October 2016–September 2017), HZ vaccinations increased by 12% compared to the prior year, from 5,146 to 5,769 doses. Influenza vaccinations increased by 37% and pertussis by 74%, while pneumococcal vaccinations decreased by 16%.

In contrast, 3 of 9 studies (33.3%) reported no improvement or a decrease in HZ vaccination rates despite pharmacist-delivered recommendation interventions. Coley et al. (17) implemented a multicomponent face-to-face and telephonic motivational interviewing program in 99 Giant Eagle pharmacies in western Pennsylvania, United States. While overall adult vaccination counts increased by 33%, HZ vaccinations decreased by 5%, attributed by the authors to the seasonal prioritization approach adopted during the project, which de-prioritized HZ messaging at certain times of year. Bacci et al. (18) deployed ImmsLink across 70 community pharmacies in Washington State; while total adult vaccinations increased by 15%, HZ vaccination “performance appeared to be unaffected” by the intervention, with no specific change in HZ vaccination counts reported. Bayraktar-Ekincioglu et al. (16) conducted individual pharmacist educational sessions in five social and solidarity centers in Ankara, Türkiye, reaching 155 participants (mean age 68.72 years). Despite 51.6% of participants expressing willingness to receive HZ vaccination following the educational session, only 0.8% (approximately 1 participant) actually received HZ vaccination at the 1-year follow-up. The HZ vaccine was not reimbursed in Türkiye at the time of the study, representing a significant cost barrier.

## 4 Discussion

This systematic review found that pharmacist-delivered recommendation strategies may improve herpes zoster vaccination uptake and RZV series completion, although the strength of the evidence remains limited by the predominance of non-randomized study designs and substantial methodological heterogeneity. More than half of the included studies reported positive vaccination outcomes (11, 13–15, 19), whereas findings from the remaining studies were inconsistent (16–18, 20).

These findings are broadly consistent with, but add important nuance to, previous systematic reviews evaluating pharmacist involvement in adult vaccination. Isenor et al. (24) conducted a systematic review and meta-analysis including 8 studies and reported that pharmacist-delivered immunization services were associated with a pooled relative risk of 1.49 (95% CI 1.09–2.04) for vaccination uptake across multiple vaccine types. Burson et al. (25) reviewed community pharmacy-based vaccination programs and concluded that pharmacies represent effective settings for adult vaccination delivery; however, they noted that the independent contribution of recommendation strategies—distinct from vaccine access, administration, and reminder systems—had not been separately quantified in that literature. Watanabe et al. (26) modeled the economic and clinical implications of increased pharmacist-facilitated HZ vaccination access and estimated meaningful public health benefits from improved pharmacist engagement. The present review extends this evidence base by focusing exclusively on HZ vaccination and specifically on the pharmacist recommendation component as the intervention. In contrast to the broader positive findings of Isenor et al. (24), only 5 of 9 studies included in the final synthesis demonstrated improvement in confirmed HZ vaccination uptake. Notably, in 6 of 9 studies in our review, pharmacist recommendations were not delivered as a standalone intervention but were embedded within multicomponent programs also including IT systems, patient reminder platforms, staff training, and motivational interviewing—therefore preventing reliable estimation of the independent effect of pharmacist recommendations.

The strongest evidence came from the cluster RCT conducted by Hess (11) which demonstrated a nearly four-fold increase in HZ vaccination rates following automated telephone recommendations (2.60% vs. 0.72%; OR 3.69; 95% CI 2.64–5.15; *p* < 0.001). Similarly, the remaining studies in which pharmacist recommendations constituted the primary intervention reported improvements in vaccination outcomes, including increased HZ vaccine uptake (11, 13) and improved RZV series completion (15).

However, these findings should be interpreted cautiously, as most available studies relied on uncontrolled pre–post designs and were susceptible to confounding from concurrent interventions, secular trends, and changes in vaccine availability. In contrast, findings from multicomponent interventions were less consistent, with some studies reporting improvements (14, 19) and others showing little or no effect on HZ vaccination outcomes (16–18). Nevertheless, the small number of studies, substantial heterogeneity in intervention design, and the predominance of non-randomized methodologies preclude firm conclusions regarding the relative effectiveness of standalone versus multicomponent recommendation strategies.

The three studies reporting no improvement or decreases in HZ vaccination rates (16–18) provide important context. The results of Coley et al. (17) highlight that in multicomponent, multi-vaccine programs, HZ vaccination may be deprioritized relative to other vaccines (e.g., influenza), particularly when seasonal messaging approaches are employed. Bacci et al. (18) similarly demonstrated that technology-supported forecasting and proactive recommendation models improved overall adult vaccination counts but did not produce a measurable increase in HZ specifically, possibly reflecting the same prioritization effect. The Turkish study by Bayraktar-Ekincioglu et al. (16) illustrates a critical contextual barrier: even when pharmacist-led educational sessions successfully improved patient willingness (51.6% of participants expressed intent to vaccinate), actual HZ vaccination remained negligible (0.8%) when the vaccine was not publicly funded and cost represented a major barrier. This finding suggests that effective recommendations alone may be insufficient to improve vaccination uptake when structural barriers, particularly vaccine cost, remain unaddressed.

The heterogeneity observed across studies suggests that the effectiveness of pharmacist-delivered recommendations may depend on the healthcare context in which they are implemented. Differences in pharmacist scope of practice, integration with immunization information systems, vaccine reimbursement policies, and the extent of proactive patient outreach may all influence intervention effectiveness.

The marked predominance of studies from the United States (8 of 9) reflects both the established role of community pharmacists as immunizers in that country and the availability of funding for pharmacy-based demonstration projects. The single Turkish study (16) highlighted distinct contextual factors—scope of practice limitations and vaccine cost/reimbursement—that substantially influence the effectiveness of pharmacist recommendation strategies.

Several important limitations of this review should be noted. First, the substantial risk of bias across the included studies, together with the near-universal absence of concurrent external control groups and the embedding of recommendation components within multicomponent interventions, makes it impossible to attribute observed changes in vaccination rates solely to pharmacist recommendations. Second, the transition from ZVL to RZV during the study period introduces heterogeneity in the outcome being measured, as RZV requires two doses and involves different administration logistics; studies conducted before October 2017 exclusively assessed ZVL, while only one study (15) explicitly evaluated RZV. Third, the diversity of outcome measures (vaccination rates, absolute counts, percentage change from prior year) and follow-up periods across studies limits direct comparison and synthesis. Fourth, the overwhelming geographic concentration in the United States limits generalizability to other healthcare systems.

Taken together, the available evidence suggests that pharmacist-delivered recommendations are a promising strategy for improving HZ vaccination outcomes, but the current evidence base remains insufficient to determine which recommendation approaches are most effective or to quantify their independent contribution to vaccination uptake.

Future research should prioritize randomized or quasi-experimental designs with concurrent control groups, clearly defined and isolated pharmacist recommendation components, and both actual vaccination uptake and series completion as primary outcomes. Studies should specify the vaccine type assessed and address the financial and regulatory context of the intervention. Research outside the United States, particularly in healthcare systems where pharmacist immunization scope of practice may be more limited, is also needed.

## 5 Conclusion

Pharmacist-delivered recommendation strategies have the potential to improve herpes zoster vaccination uptake and support completion of the recombinant zoster vaccine series. The available evidence suggests that proactive outreach strategies, particularly telephone-based recommendations and follow-up, may be effective approaches to increasing HZ vaccination uptake and RZV series completion. However, the certainty of the evidence remains limited due to the predominance of non-randomized studies, substantial methodological heterogeneity, and the frequent use of multicomponent interventions. Further rigorously designed studies are needed to determine the independent effectiveness of pharmacist-delivered vaccination recommendations and to identify the most effective recommendation strategies.
