# Supplementary material for: Effectiveness of pharmacist-delivered vaccination recommendations for herpes zoster: a systematic review
Source: Front Public Health. 2026 Aug 17;14:1884479. doi: 10.3389/fpubh.2026.1884479 (PMC13526920; doi:10.3389/fpubh.2026.1884479)
Supplement: Supplementary file 1 [file Supplementary_file_1.docx]

**Supplementary Material 1. Database search strategies**

**Table S1. Search strategy – The Cochrane Library**

| **ID** | **Search** | **Hits (16.10.2025)** |
| --- | --- | --- |
| #1 | (pharmacist):ti,ab,kw | 6 724 |
| #2 | community pharmacy | 2 639 |
| #3 | pharmaceutical care | 11 000 |
| #4 | pharmaceutical service | 2 212 |
| #5 | #1 OR #2 OR #3 OR #4 | 21 057 |
| #6 | shingles | 251 |
| #7 | herpes zoster | 3 006 |
| #8 | postherpetic | 1 434 |
| #9 | post-herpetic | 423 |
| #10 | #6 OR #7 OR #8 OR #9 | 3 916 |
| #11 | #5 AND #10 | 130 |

**Table S2. Search strategy – Embase**

| **ID** | **Search** | **Hits (16.10.2025)** |
| --- | --- | --- |
| #1 | 'pharmacist'/exp OR 'pharmacist' | 136 816 |
| #2 | community AND pharmacy | 65 892 |
| #3 | pharmaceutical AND care | 165 152 |
| #4 | pharmaceutical AND service | 50 985 |
| #5 | #1 OR #2 OR #3 OR #4 | 336 927 |
| #6 | herpes AND zoster | 46 214 |
| #7 | shingles | 3 416 |
| #8 | postherpetic | 9 072 |
| #9 | 'post herpetic' | 2 231 |
| #10 | #6 OR #7 OR #8 OR #9 | 51 279 |
| #11 | #5 AND #10 | 728 |
| #12 | #11 AND 'article'/it | 367 |

**Table S3. Search strategy – PubMed**

| **ID** | **Search** | **Hits (16.10.2025)** |
| --- | --- | --- |
| #1 | pharmacist | 58 588 |
| #2 | "community pharmacy" | 53 240 |
| #3 | "pharmaceutical care" | 167 994 |
| #4 | "pharmaceutical service*" | 93 879 |
| #5 | #1 OR #2 OR #3 OR #4 | 272 768 |
| #6 | "herpes zoster" | 18 955 |
| #7 | shingles | 22 547 |
| #8 | postherpetic | 4 256 |
| #9 | "post-herpetic" | 1 232 |
| #10 | #6 OR #7 OR #8 OR #9 | 24 293 |
| #11 | #5 AND #10 | 339 |

Full PubMed search string (as of 16.10.2025)

( "pharmacist's"[All Fields] OR "pharmacists"[MeSH Terms] OR "pharmacists"[All Fields] OR "pharmacist"[All Fields] OR ("pharmacies"[MeSH Terms] OR "pharmacies"[All Fields] OR ("community"[All Fields] AND "pharmacy"[All Fields]) OR "community pharmacy"[All Fields]) OR ("pharmaceutical services"[MeSH Terms] OR ("pharmaceutical"[All Fields] AND "services"[All Fields]) OR "pharmaceutical services"[All Fields] OR ("pharmaceutical"[All Fields] AND "care"[All Fields]) OR "pharmaceutical care"[All Fields]) OR (("biopharmaceutics"[MeSH Terms] OR "biopharmaceutics"[All Fields] OR "pharmaceutic"[All Fields] OR "pharmaceutics"[All Fields] OR "pharmaceutical preparations"[Supplementary Concept] OR "pharmaceutical preparations"[All Fields] OR "pharmaceutical"[All Fields] OR "pharmaceutical preparations"[MeSH Terms] OR ("pharmaceutical"[All Fields] AND "preparations"[All Fields]) OR "pharmaceuticals"[All Fields] OR "pharmaceutical's"[All Fields] OR "pharmaceutically"[All Fields]) AND "service*"[All Fields])) AND ("herpes zoster"[All Fields] OR ("herpes zoster"[MeSH Terms] OR ("herpes"[All Fields] AND "zoster"[All Fields]) OR "herpes zoster"[All Fields] OR "shingles"[All Fields] OR "shingle"[All Fields]) OR "postherpetic"[All Fields] OR "post-herpetic"[All Fields])

## **Table S4. Risk of Bias Assessment**

## **Herpes Zoster Vaccination Intervention Studies**

| Study (first author, year) | Design / comparator | Selection bias | Information bias | Confounding | Overall risk of bias |
| --- | --- | --- | --- | --- | --- |
| Bedwick, 2017 | Single pharmacy pre–post with historical control | High – single site; no randomization; historical comparison | Low – vaccination counts from pharmacy records | High – secular trends and other promotional activities not controlled | Serious |
| Hess, 2013 | Cluster RCT (8 intervention vs 8 control pharmacies) | Some concerns – limited detail on allocation concealment | Low – objective vaccination counts from pharmacy system | Some concerns – other local campaigns not controlled | Some concerns |
| Wang, 2013 | 3 pharmacies; pre–post with different calendar months | High – non-randomized; seasonal differences possible | Low – vaccination counts from pharmacy records | High – concurrent campaigns and other influences not controlled | Serious |
| Tyler, 2021 | Retrospective cohort in 10 pharmacies | Some concerns – non-randomized; patients receiving calls may differ | Low – completion measured from dispensing records | High – limited adjustment for confounders | Serious |
| Bayraktar-Ekincioglu, 2022 | Pre–post cohort with 1-year follow-up | High – volunteer participants; single education session | Some concerns – vaccination self-reported by phone | High – multiple external influences not controlled | Critical |
| Coley, 2020 | Multi-site pre–post project (99 pharmacies) | Some concerns – no randomized control; population changes possible | Low – vaccination counts from pharmacy systems | High – secular trends not controlled | Serious |
| Bacci, 2019 | Multi-site pre–post project (70 pharmacies) | Some concerns – no concurrent control group | Low – vaccination counts from pharmacy systems | High – concurrent system and policy changes not controlled | Serious |
| Wehbi, 2019 | Multi-site pre–post project (159 pharmacies) | Some concerns – no control group; between-year differences | Low – vaccination counts from pharmacy records | High – other vaccination initiatives not controlled | Serious |
| Lin, 2018 | Pre–post study with two non-randomized training approaches | High – purposive site selection; non-randomized training allocation | Some concerns – HZ vaccination not reported separately | High – concurrent influences not controlled | Serious |

## Abbreviations: RCT, randomized controlled trial.
